# Functional consequences of Palaeozoic reef collapse

**DOI:** 10.1038/s41598-022-05154-6

**Published:** 2022-01-26

**Authors:** Tom C. L. Bridge, Andrew H. Baird, John M. Pandolfi, Michael J. McWilliam, Mikołaj K. Zapalski

**Affiliations:** 1 Australian Research Council Centre of Excellence for Coral Reef Studies, James Cook University, Townsville, QLD 4811 Australia; 2 Biodiversity and Geosciences Program, Museum of Tropical Queensland, Queensland Museum Network, Townsville, QLD 4810 Australia; 3 School of Biological Sciences and Australian Research Council Centre of Excellence for Coral Reef Studies, The University of Queensland, St Lucia, QLD 4067 Australia; 4 Hawaii Institute of Marine Biology, University of Hawaii at Manoa, Kaneohe, HI 96744 USA; 5 Faculty of Geology, University of Warsaw, 02-089 Warsaw, Poland

**Keywords:** Climate-change ecology, Palaeoecology

## Abstract

Biogenic reefs have been hotspots of biodiversity and evolutionary novelty throughout the Phanerozoic. The largest reef systems in Earth’s history occurred in the Devonian period, but collapsed during the Late Devonian Mass Extinction. However, the consequences for the functional diversity of Palaeozoic reefs have received little attention. Here, we examine changes in the functional diversity of tabulate coral assemblages over a 35 million year period from the middle Devonian to the Carboniferous, straddling the multiphase extinction event to identify the causes and ecological consequences of the extinction for tabulate corals. By examining five key morphological traits, we show a divergent response of taxonomic and functional diversity to the mass extinction: taxonomic richness peaked during the Givetian (~ 388–383 Ma) and coincided with peak reef building, but functional diversity was only moderate because many species had very similar trait combinations. The collapse of taxonomic diversity and reef building in the late Devonian had minimal impact on functional richness of coral assemblages. However, non-random shifts towards species with larger corallites and lower colony integration suggest a shift from photosymbiotic to asymbiotic taxa associated over the study period. Our results suggest that the collapse of the huge Devonian reef systems was correlated with a breakdown of photosymbiosis and extinction of photosymbiotic tabulate coral taxa. Despite the appearance of new tabulate coral species over the next 35 million years, the extinction of taxa with photosymbiotic traits had long-lasting consequences for reef building and, by extension, shallow marine ecosystems in the Palaeozoic.

## Introduction

Biogenic reefs built by calcifying sessile benthos, such as corals, have been hotspots of biodiversity and origination throughout the Phanerozoic^1^. However, reef growth through time shows strong ‘boom and bust’ cycles, attributable to both changes in environmental conditions and biological factors^2^. The loss of calcifying marine invertebrates can lead to the cessation of carbonate production, known as ‘reef crises’, which are often, but not always, associated with mass extinctions^3,4^. Contemporary climate change is predicted to dramatically alter the diversity and ecological function of reef ecosystems; therefore, understanding the ecological consequences of past reef crises provides important insights into the fate of contemporary reef systems^5^.

**Figure 1:**
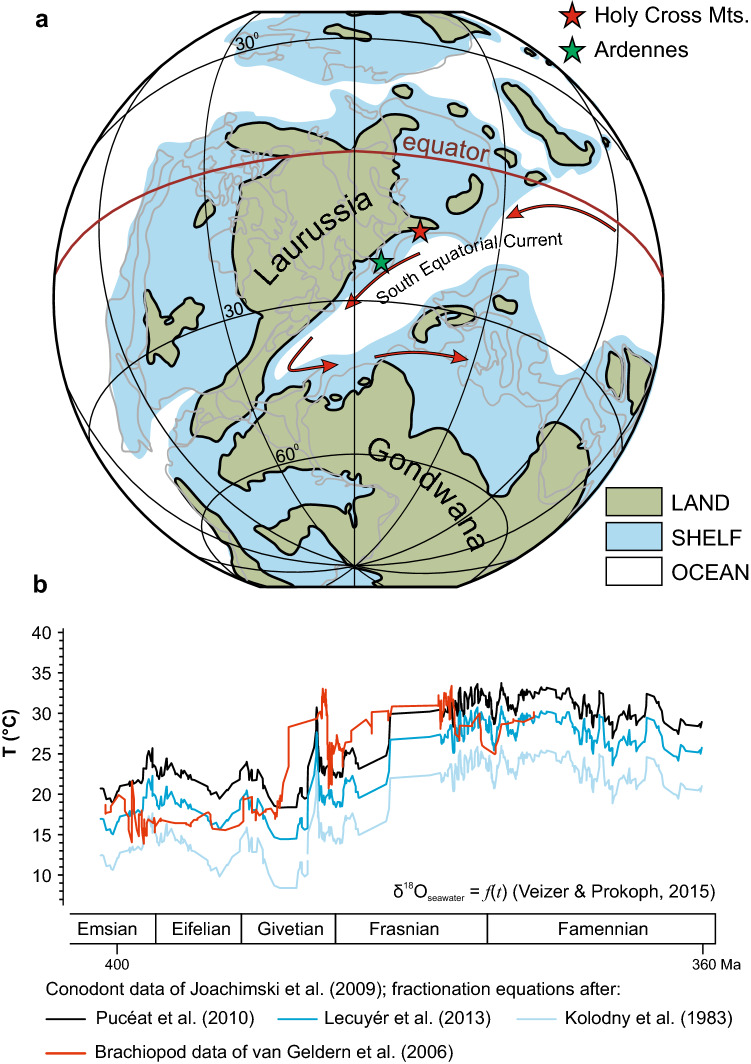
Location of Ardennes and the Holy Cross Mountains on the Devonian palaeogeographic map (**a**), redrawn from Jakubowicz et al. (2019); changes in temperatures across the Middle and Late Devonian (**b**), redrawn from Zapalski et al. (2017)^11^. Palaeogeographic map originally based on Scotese (2001).

The Devonian period of the Palaeozoic Era (~ 419–359 Ma) saw the largest development of metazoan reefs in the Earth’s history^6,7^. Unlike contemporary coral reefs, Devonian reefs occurred in a ‘super greenhouse’ world with high calcite, and low aragonite seawater chemistry^8,9^^.^ Although temperatures did vary considerably across the Devonian (Fig. 1B), the period was likely characterised by minimal temperature gradients from the equator to the poles and a corresponding lack of polar ice caps. Tropical sea temperatures rose rapidly to at least ~ 28 °C^10^ and potentially > 32 °C in the latest Givetian (upper bound: ~ 383 Ma), followed by a rapid decrease in the early Frasnian^11^. Temperature rose steadily through the Frasnian and again exceeded at least 32 °C in the latest Frasnian (upper bound: ~ 388 Ma) before decreasing again in the Famennian^10,11^. CO_2_ levels were substantially higher than the present, probably approaching 2000 ppm for much of the Devonian^10,12,13^, resulting in “calcite seas” with a Mg/Ca ratio < 2^9^.

Despite high atmospheric CO_2_ and ‘super greenhouse’ climate, corals and hypercalcifying stromatoporoid sponges constructed vast barrier reef tracts even larger than their contemporary analogues^3,14^. Devonian reefs and bioconstructions occur in many parts of the world, including Europe, North America, Africa and Australia. While detailed geomorphic and sedimentological studies have been conducted on Devonian reefs from North America and Australia, from a taxonomic perspective the best-studied reefs are those of the Ardennes (Belgium and France) and the Holy Cross Mountains (Poland) in northern Europe^15^. These sites form part of a large fossil reef system extending from England to Poland that during the Devonian were located ~ 1000 km apart on the tropical southern shelf of Laurussia (Fig. 1a)^8,16,17^. The arrangement of the reef tract reflects the palaeogeography of the southern margin of Laurussia, bordering the Variscan Sea (or Rheic Ocean)^18^. The seaway between Laurussia (in the north) and Gondwana (in the south) received westward-flowing warm waters from the South Equatorial Current^19–21^, which facilitated dispersal of benthic organisms and resulted in homogeneous ecological communities along this shelf. These huge reef systems collapsed during the Late Devonian Mass Extinction (*ca.* 372 Ma)^7,22^, one of the ‘Big Five’ extinction events of the Phanerozoic, which strongly affected many groups of benthic marine invertebrates^23–25^.

Tabulate corals are an extinct subclass of anthozoans with calcite skeletons that occurred throughout most of the Palaeozoic. They had a wide range of growth forms rivalling that of modern Scleractinia, from fine-branching (e.g. *Striatopora, Coenites*) to foliose (e.g*. Platyaxum, Alveolites*) to massive and sub-massive (e.g. *Favosites, Heliolites*). This morphological diversity allowed tabulate corals to dominate a wide range of reef habitats, from mesophotic reef habitats to shallow turbid bays^26,27^. Both tabulate corals and hypercalcifying stromatoporoid sponges reached their highest taxonomic diversity coincident with the period of maximal reef development in the Givetian stage of the Middle Devonian^7,8,28^. Their diversity declined substantially during the Late Devonian Mass Extinction, although sparse tabulate coral faunas persisted throughout the latest Devonian (Famennian)^29–31^. By the Early Carboniferous tabulates were once again contributing to reef building, but never regained their dominance.

Photosymbiosis is proposed as a key factor to facilitate reef building, and reef crises are often linked to a breakdown in photosymbiosis^3,32^. There is evidence that at least some tabulate corals were photosymbiotic^8,33–35^, which may have contributed to both their capacity to build extensive reefs in the Middle Devonian and the collapse of reef building during the Late Devonian. The decline in tabulate corals and associated reef collapse in the late Givetian (particularly the Taghanic event, one of multiple extinction ‘pulses’ in the late Devonian) and also at the Frasnian/Famennian boundary (Kellwasser events) coincided with spikes in sea surface temperatures (late Givetian) and a more gradual rise in the late Frasnian (Late Devonian Hothouse)^10,11^. Although these events occurred across longer timescales, the collapse of tabulate corals during the Late Devonian mass extinction provides an historical analogue to the long-term effects of contemporary climate change, particularly thermal stress, on reef ecosystems^11^.

Studies of modern scleractinian corals demonstrate that certain traits, including corallite size, the level of colony integration (a measure of the isolation of individual corallites within the colony) and skeletal porosity, can be used to infer the likelihood of whether a species was photosymbiotic^33,36,37^. Non-symbiotic corals are characterised by non-porous skeletons, low colony integration and large corallite size (> 1.3 mm), and never display growth bands in macroscale^11^. Therefore, fossil corals with highly perforated skeletons, very small (submillimetric) corallites and clear growth banding were almost certainly photosymbiotic. Although colony integration was somewhat lower in tabulates than in modern scleractinians, they nonetheless showed considerable variation in colony integration among species^11^. Therefore, examining changes in the morphological trait space of tabulate corals through time could provide insight into ecological effects of the late Devonian mass extinctions on Palaeozoic reef communities. Here, we test for non-random shifts in the morphological trait space occupied by tabulate coral assemblages through time in an attempt to shed light on the likelihood of a shift from photosymbiotic to non-symbiotic taxa coinciding with reef collapse in the Late Devonian (see^11^).

The reef tract along the south-eastern flank of the Eastern Laurussia is one of the most typical, and best recognized reef belts of the Middle-Late Devonian, and appears representative of Devonian reefs on tropical Rheic shelves^8,38^. It is also the most complete taxonomic record of reefs from this time. These locations therefore provide an ideal case study to examine changes in morphological traits of tabulate coral assemblages in response to climatic fluctuations through the Devonian and early Carboniferous. Here, we quantify morphological traits of tabulate coral assemblages across the Devonian and early Carboniferous to examine changes in the functional diversity of coral assemblages through time, spanning two ‘pulses’ associated with the late Devonian mass extinction. Specifically, we aim to identify (1) how functional richness of tabulate coral assemblages varied through time; (2) whether there was a relationship between taxonomic and functional richness in tabulate coral assemblages; and (3) whether tabulate corals had non-random responses to extinction events that provide insight into the causes of reef collapse in the Palaeozoic.

## Results

**Figure 2:**
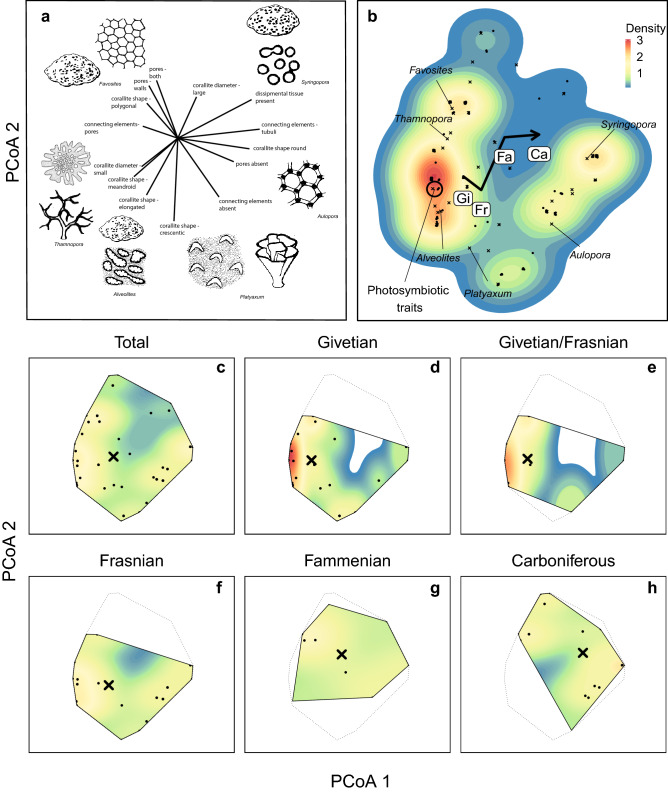
Trait space for Devonian and early Carboniferous tabulate corals; (**a**) Vectors associated with trait space for Devonian tabulates, showing features of corals associated with each part of the trait space; (**b**) trait space for the entire study period. Colours indicate the density of species (i.e. functional redundancy) in each area of trait space. Letters indicate the centroid of the trait space in each time period: Gi = Givetian, Fr = Frasnian, Fa = Fammenian; Ca = Carboniferous; (**c**–**h**) functional richness of tabulate corals during each time period relative to the total for the entire study period. Dotted lines represent the proportion of the total traitspace occupied in each time period. Points represent the location of each species in the trait space Crosses indicate the centroids of each genus. The most dominant genera are named to indicate their location in the traitspace.

The first two principal component axes were broadly associated with colony integration (connecting elements, presence/absence of pores and dissepimental tissues) and corallite diameter, respectively (Fig. 2a). Species in genera with low colony integration (e.g., *Aulopora, Syringocystis, Maksymilianites, Syringopora*) occur on the right of the traitspace, while those on the left represent species with high colony integration (e.g., *Alveolites, Scoliopora, Coenites, Platyaxum*) (Table S1). Axis 2 corresponds to corallite diameter – taxa with small corallites occur towards the bottom, and large corallites towards the top of the trait space (Fig. 2a,b). All five traits were significantly correlated (Pr > r = < 0.001) with the ordination of species in multivariate space in the principal coordinates analysis (Table 1). Two morphological traits alone, corallite shape and the presence of connecting elements, explained > 70% of the total variance in the ordination.

**Table 1:** Correlation of each trait to the principal coordinate ordination based on 999 random permutations.

Morphological trait	r^2^	Pr(> r)
Corallite diameter	0.45	0.001
Dissipmental tissue	0.42	0.001
Connecting elements	0.70	0.001
Pores	0.69	0.001
Corallite shape	0.74	0.001

**Figure 3:**
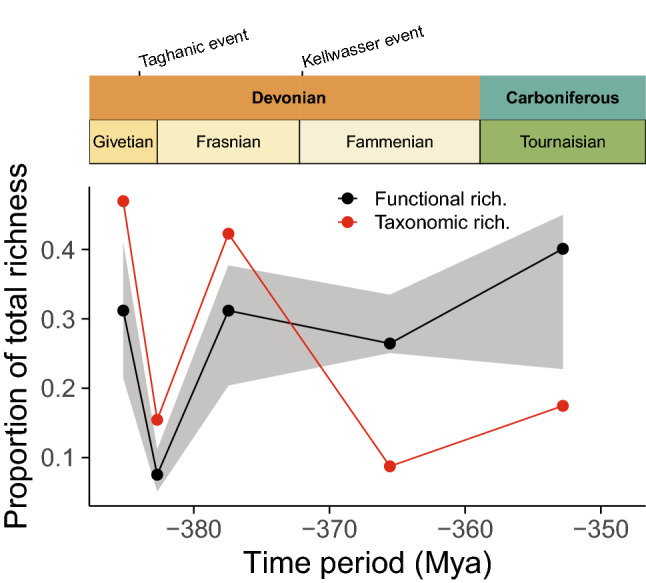
Functional and taxonomic richness of tabulate coral assemblages from the Givetian to the Carboniferous. Grey ribbon indicates 95% confidence intervals of the trait sensitivity analysis.

Functional richness of tabulate coral assemblages varied considerably over the 35 million years from the Middle Devonian to the Early Carboniferous and straddling the Late Devonian reef crisis. Taxonomic richness was highest in the Givetian, coinciding with the peak of reef building, but collapsed at the Givetian/Frasian boundary during the Taghanic event (Fig. 3). Taxonomic richness recovered substantially during the Frasnian due to the emergence of new species, before collapsing again into the Fammenian (Kellwasser events), and remained relatively low in the Carboniferous. Functional diversity was high in the Givetian before collapsing at the Givetian/Frasnian boundary. However, unlike taxonomic richness, functional richness recovered during the Frasnian, and remained relatively high in the Fammenian before increasing further in the Carboniferous. Despite low taxonomic richness, Fammenian and Carboniferous tabulate coral assemblages occupied a similar (Fammenian) or greater (Carboniferous) proportion of the trait space than assemblages in the peak of reef building during the Givetian.

We also found considerable differences in the areas of the trait space occupied through time. During the Givetian period when reef building was high, tabulate coral assemblages were heavily dominated by species with small corallites and porous skeletons (left side of the trait space (Fig. 2a,b,d), while taxa with corallites > 1 mm were rare. The high density of species in this region of trait space shows that although Givetian assemblages were richer in species than at any other time, many species had very similar traits and potentially similar ecological roles (i.e., high functional redundancy).

**Figure 4:**
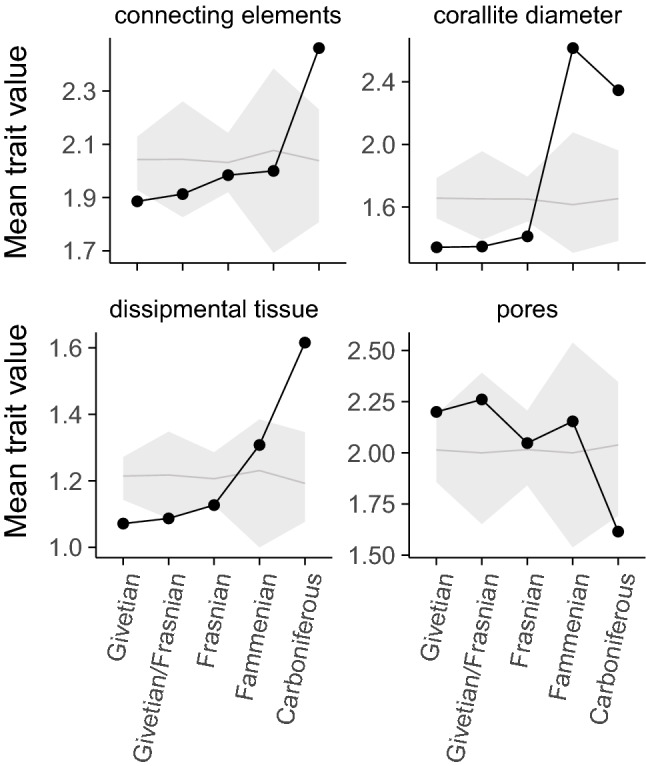
Directional change in mean trait values for ordered categorical traits through time. Grey lines indicate expected trait means based on random permutations. Movement outside the grey ribbon indicates non-random change, and a point outside the ribbon shows that trait values of the assemblage at that time are more distinct that 95% of the random expectations. Trait values refer to the character states in Table S2.

The Taghanic event at the Givetian/Frasnian boundary disproportionately affected species with small corallites and highly integrated skeletons at the bottom of the traitspace (Fig. 2e). For example, branching *Thamnopora* with small corallites went extinct, while *Thamnopora* spp. with large corallites (> 2 mm in diameter) survived. From the Frasnian through the Fammenian and into the Carboniferous, tabulate coral assemblages are increasingly composed of taxa with large corallites and low colony integration (upper-right area of the trait space; Fig. 2f–h). The area of trait space associated with small corallites and high colony integration is represented by just a single known specimen (Alveolitidae indet.) in the Famennian^31^, and Carboniferous corals are similarly characterized by very large corallites which are virtually unknown in earlier faunas. The small number of species occupying large areas of trait space indicates that Late Devonian and Carboniferous assemblages exhibited low functional redundancy (Fig. 2c-h). The null model analysis also shows that the directionality of change in morphological traits was not random (Fig. 4). Across the assemblage there are non-random shifts in colony integration from pores to connecting tubes, towards larger corallite sizes and an increase in the prevalence of dissipmental tissues, particularly in the Fammenian and Carboniferous.

## Discussion

The late Devonian reef crises had a substantial impact on the taxonomic and functional richness of Palaeozoic coral assemblages. However, our data suggest a decoupling of taxonomic and functional richness in the latest Devonian and early Carboniferous. Givetian assemblages were characterised by a large number of species with small corallites and high colony integration (as defined by^11^); however, many species with these traits went extinct in the end-Givetian extinction events, which also coincided with the collapse of the large Devonian reef systems. Importantly, species with trait combinations common in Givetian tabulate corals did not re-appear for at least 35 million years. While tabulate corals with larger corallites occurred in low abundance throughout the Late Devonian and Carboniferous, reef building remained depressed relative to the mid-Devonian. While reef-like structures dominated by metazoans other than corals (sponges, bryozoans) persisted in the Carboniferous and Permian, corals did not function as the primary reef-builders at least until the rise of the Scleractinia in the mid-Triassic^22^.

While coral assemblages in the latest Devonian and Carboniferous occupied a relatively large proportion of functional space they were far less taxonomically diverse than those in the Givetian. In contrast, the trait space in the Givetian contains many species with very similar traits on the left hand side of the trait space. The loss of taxa with the particular trait combinations commonly present during peak reef building in the Givetian suggests that these traits were closely associated with reef-building, and that their loss was correlated with the collapse of Devonian reefs. However, the strongest signal of non-random change in functional diversity occurs in the Fammenian and Carboniferous. The most likely explanation for this pattern is that the high-diversity assemblages of the Givetian exhibited relatively high functional redundancy, where many species could be lost with only minor impact on overall functional diversity. However, in the depauperate assemblages of the latest Devonian and Carboniferous, the loss of only a single species could have a large influence on functional diversity. Consequently, functional redundancy in high-diversity assemblages may have led to some level of response diversity and obscured the signal of the non-random shifts in trait composition during the Taghenic event. Nonetheless, the changes in the density of species across the trait space clearly demonstrate a loss of species with certain trait combinations occurred at the Givetian/Frasnian boundary.

The traits associated with Givetian tabulates are commonly associated with photosymbiosis in modern corals, and therefore our results support the hypothesis^3,11^ that the collapse of Devonian reefs may have been associated with a breakdown of photosymbiosis. We observed non-random shifts in all four of the ordered categorical traits examined through the 35 million year study period, particularly following the Kellwasser events at the end of the Frasnian period. The shift in connecting elements from pores to tubes signifies a decrease in colony integration. Increased presence of dissipmental tissues is associated with corals occurring in environments with higher sedimentation rates, and combined with increased corallite size suggests a shift towards environments less suitable for photosymbiosis and characters associated with heterotrophic feeding^39^. Although tabulate coral assemblages exhibited a wide range of trait combinations following the Givetian, few species possessed the small corallites and high colony integration associated with peak reef building, and tabulate corals never regained the capacity to build reefs. Consequently, our results suggest that the loss of only a relatively small proportion of trait space, associated with species with the capacity for photosymbiosis, were integral to the construction of Devonian reefs.

The late Givetian events (principally the Taghanic event, but also later, minor pulses (see^40^) were caused primarily by the rise of sea temperatures and sea level, with additional factors including changes in the carbon cycle and increased nutrient input, while reduced oxygenation in epicontinental seas particularly affected benthic communities^41,42^. Palaeo-SST during the Givetian Thermal Maximum (385–382 Ma^10^) rose rapidly to at least 27.8 °C^10^ and potentially exceeded 32 °C^11^ in the tropical zone, and were among the highest temperatures of the entire Devonian. Also temperatures by the end of Frasnian (Kellwasser Thermal Maximum, 372.5 Ma^10^) exceeded 32°C^11^ or even 33°C^10^. On modern reefs, breakdown of photosymbiosis occurs when sea surface temperatures exceed the mean monthly maximum temperature for an extended period. Although the bleaching threshold varies considerably in space due to adaptation of corals to their local thermal history, temperatures in excess of 31 °C are generally sufficient to induce bleaching in all but the most warm-adapted coral assemblages^43^.

One of the triggers of global climatic changes by the end of Devonian, particularly the Kellwasser, was increased Eovariscan volcanic activity^44^ which likely contributed to substantial rises in temperature. Although the timescales are clearly very different, and the uncertainties associated with estimating Devonian SSTs, the multiple periods of high sea temperatures during the Middle-Late Devonian and the resulting collapse of reef building and declines of hypercalcifying organisms may be linked to temperature-related breakdown of photosymbiosis. While ocean acidification is one potential threat to modern reefs dominated by aragonitic Scleractinia, it appears to have had a negligeable effect on Devonian reefs^45^, although anoxia and other factors may have locally played some role in the extinctions of tabulate corals and associated reef collapse (see^11^). Taxa with small corallites and high colony integration – characteristics that are very rare in non-photosymbiotic corals today—were highly abundant during peak reef building in the Givetian, but strongly affected by the late Givetian events. Taxa with similar traits reappeared in the Frasnian but were extinct by the Fammenian. Colonies with plating morphology, generally considered as photosymbiotic^34^ were also absent after the Givetian. The congruence between the effects of high temperatures and functional changes in coral assemblages separated by 380 million years provides evidence that a breakdown of photosymbiosis may have been responsible for the collapse of the massive reef systems of the middle Devonian.

There are a number of caveats that must be considered when comparing functional change in Devonian versus modern reefs, particularly the temporal scale over which changes are assessed. Temporal ranges of taxa vary, and despite the excellent preservation and extensive examination of the fossil corals in the Ardennes and Holy Cross Mountains, the stratigraphic ranges of species are at stage resolution (~ 10 Ma) and therefore strongly time-averaged. The duration of any given taxon was usually shorter than the stratigraphic range recorded here, but the true species durations are obscured by the temporal resolution of stratigraphic data. Nonetheless, given that we are investigating the role of corals in building the reef tract and that the duration of this process was longer than the stages themselves (Givetian – 5 Ma, Givetian-Frasnian reef existence—~ 15 Ma), time-averaging should not affect our conclusions^46^.

Given the interest in the effects of environmental change on ecosystem function, this study provides new insight into the long-term consequences of a severe biological crisis on ancient reef ecosystems. There are clearly numerous differences between the collapse of reef systems in the Middle-Late Devonian and the modern reefs: unlike today, Devonian reefs occurred during a period of high atmospheric carbon dioxide and calcite seas; tabulate corals grew calcitic instead of aragonitic skeletons, and the rates of change in the Devonian were likely considerably slower than those observed today. Nonetheless, we provide evidence that the Middle-Late Devonian reef crisis resulted in the non-random extinction of species with trait combinations similar to those of photosymbiotic reef-building scleractinian corals today. Importantly, we show that the effects of the late reef collapse persisted for at least 35 million years but likely far longer, as extensive coral reef systems did not appear again until the Scleractinian reefs of the Middle Triassic 150 million years later. Although new species of tabulate corals emerged, and the group persisted until the end-Permian mass extinction 252 million years ago, their traits differed from species from the Givetian, and they never again developed the ability to build extensive reef systems. These results demonstrate that the events that precipitated the collapse of Devonian reefs, and the likely breakdown of photosymbiosis in tabulate corals, had important legacy effects on shallow marine ecosystems extending tens of millions of years beyond the extinction events. This finding has important implications for the understanding the future of contemporary coral reef biodiversity. Given the importance of reefs to shallow marine biodiversity in ancient and modern seas, the collapse of tropical shallow-water reef ecosystems due to anthropogenic climate change could alter the functioning of marine ecosystems for millions of years.

## Methods

We quantified the tabulate coral assemblages in each of four time periods from the Middle to Late Devonian (Givetian, Frasnian and Famennian) to the Early Carboniferous. This ~35 million year period spans the peak of reef building in the Middle Devonian the late Devonian mass extinction, which we define as including both the Taghanic and Kellwasser events. We use the term Late Devonian mass extinction (with an upper case L) refers only to Kellwasser events, which occurred during the formal time unit ratified by the International Commission on Stratigraphy; in contrast, late Devonian with a lower case L simply refers to the later part of the Devonian. We distinguished five tabulate assemblages across this time period: Givetian, Givetian/Frasnian boundary (survivors of the late Givetian events), Frasnian, Famennian (survivors of the Kellwasser events) and the Early Carboniferous. A total of 157 species of tabulate corals were recorded from Ardennes and the Holy Cross Mountains during the study period. The species list for the Devonian was extracted from^11^, which presents the most complete list while also verifying the presence and taxonomic identification of the taxa from previous studies^47,48^. Carboniferous assemblages, which were not included in^11^, were extracted from^40,49^, and include the taxa described from the Holy Cross Mountains and a nearby Cracow area locality. Taxonomic richness was defined as the number of species recorded as occurring in a stage. Eight species were excluded from the analysis due to incomplete trait data, leaving 149 species in our analysis (Table S1).

We quantified five morphological traits that could be measured effectively from fossil tabulate corals: presence of dissipmental tissue, type of connecting elements, presence and location of pores, corallite shape, and corallite diameter (Table S2). Traits selected were based on their potential to indicate differences in life history strategies among species, and as much as possible were analogous to life history traits examined using modern corals^50^. All traits were converted to ordered categorical variables with multiple levels prior to analysis, with the exception of corallite shape. Traits associated with colony integration were ordered from least integrated to most highly integrated, and corallite sizes from small to large (Table S2). Multivariate distance between each species was calculated using a principal coordinate analysis (PCoA) based on a Gower distance matrix using the R functions ‘gowdis’ (‘FD’ package^51,52^) and ‘cmdscale’ (‘stats’ package). The functional diversity of the tabulate coral assemblage in each time period was calculated as the four-dimensional convex hull volume of trait space using the packages FSECchange^53^ in R. The proportion of the total trait space occupied in each time period (defined as functional richness by Mouillot et al.^53^) was calculated by dividing the volume of the convex hull for each time period by the total convex hull (including all species recorded in the study across all time periods). We also calculated the centroid of the trait space for the entire study period and each of the five time periods to test for shifts in the mean distribution of traits within each assemblage.

The correlation between each morphological trait and the ordination in multivariate space was examined using 999 permutations in the ‘envfit’ function from the R package ‘vegan’^54^. To allow comparison between taxonomic and functional diversity in each time period, both variables were expressed as the proportion relative to the total for the entire study period (e.g., richness = richness for each time period / richness for the entire study period). To test how arbitrary variations in the trait-based analyses influenced our results, we conducted a sensitivity analysis by repeating our calculations of functional richness multiple times with each trait removed prior to constructing the PCoA axes. To test for non-random trait change over time, we compared average trait values for each of the ordered categorical traits (i.e. excluding corallite shape) in each period with a null model of random species selection, while keeping species richness constant across the time periods. Random selections were done 1000 times for each period.

## Supplementary Information

Supplementary Table S1.

Supplementary Table S2.
