# Author Correction: Functional consequences of Palaeozoic reef collapse

**DOI:** 10.1038/s41598-022-08420-9

**Published:** 2022-03-15

**Authors:** Tom C. L. Bridge, Andrew H. Baird, John M. Pandolfi, Michael J. McWilliam, Mikołaj K. Zapalski

**Affiliations:** 1 Australian Research Council Centre of Excellence for Coral Reef Studies, James Cook University, Townsville, QLD 4811 Australia; 2 Biodiversity and Geosciences Program, Museum of Tropical Queensland, Queensland Museum Network, Townsville, QLD 4810 Australia; 3 School of Biological Sciences and Australian Research Council Centre of Excellence for Coral Reef Studies, The University of Queensland, St Lucia, QLD 4067 Australia; 4 Hawaii Institute of Marine Biology, University of Hawaii at Manoa, Kaneohe, HI 96744 USA; 5 Faculty of Geology, University of Warsaw, 02-089 Warsaw, Poland

Correction to: *Scientific Reports* 10.1038/s41598-022-05154-6, published online 26 January 2022

The Supplementary Information file published with this Article contained errors.

In Table S1, the data for columns “Givetian”, “Givetian/Frasnian”, “Frasnian”, “Fammenian” and “Carboniferous” was omitted. As a result, the Supplementary Information file has been split into two separate files: Supplementary Table S1 and Supplementary Table S2.

The original Supplementary Information file is provided below.

This error has now been corrected in the Supplementary Information files that accompany the original Article.

## Supplementary Information

Supplementary Information.

