# Supplementary material for: Functional consequences of Palaeozoic reef collapse
Source: Sci Rep. 2022 Jan 26;12:1386. doi: 10.1038/s41598-022-05154-6 (PMC8792005; doi:10.1038/s41598-022-05154-6)
Supplement: Supplementary file 2 — Supplementary Table S2. [file 41598_2022_5154_MOESM2_ESM.docx]

**Table S2.** Morphological characters, character states, and interpretations of ecological significance of each morphological character. Additional references not cited in the main text are provided below.

| **Morphological Character** | **Character States** | **Interpretation** | **Source** |
| --- | --- | --- | --- |
| dissepimental tissue | 1-absent, 2-present | Feeding strategy, sediment rejection. Dissipmental tissue present indicates adaptation to turbid environments | Berkowski 2012, Sorauf 2007 |
| connecting elements | 1-absent, 2-pores; 3-tubuli | Increasing colony integration and likelihood of photosymbiosis | Hughes 1987  [11, 33,36,37]. |
| pore location | 1-absent, 2-wall, 3-corners, 4 both | Colony-wide coordinated response to stress, likelihood of photosymbiosis, | Swain et al. 2018  [33,36,37]. |
| corallite shape | 1-round; 2-subpolygonal/polygonal; 3-elongated; 4-meandroid, 5-crescentic | Colony integration |  |
| corallite diameter | 1 – small (<1mm), 2 – medium (1-2mm), 3 –large (>2mm) | Likelihood of photosymbiosis as only photosymbiotic corals possess small corallites. | Porter et al. 1976 |

**References**

Berkowski, B. 2012. Life strategies and function of dissepiments in rugose coral *Catactotoechus instabilis* from the Lower Devonian of Morocco. Acta Palaeontologica Polonica 57 (2): 391–400.

Hughes, T. P. (1987). Skeletal density and growth form of corals. *Marine Ecology Progress Series*, 35, 259-266.

Porter, J.W. (1976) Autotrophy, heterotrophy, and resource partitioning in Caribbean reef-building corals. *The American Naturalist* 110:731–742.

Sorauf, J. E. 2003. The function of dissepiments and marginaria in the Rugosa (Cnidaria, Zoantharia). *Fossil Corals and Sponges, Proceedings of the 9th International Symposium on Fossil Cnidaria and Porifera, Graz*, 11-29.

Swain, T. D., Bold, E. C., Osborn, P. C., Baird, A. H., Westneat, M. W., Backman, V., & Marcelino, L. A. (2018). Physiological integration of coral colonies is correlated with bleaching resistance. *Marine Ecology Progress Series*, 586, 1-10.
