# Supplementary material for: Author Correction: Functional consequences of Palaeozoic reef collapse
Source: Sci Rep. 2022 Mar 15;12:4429. doi: 10.1038/s41598-022-08420-9 (PMC8924185; doi:10.1038/s41598-022-08420-9)
Supplement: Supplementary file 1 — Supplementary Information. [file 41598_2022_8420_MOESM1_ESM.docx]

Table S1. Species examined in this study and their associated traits. Numbers refer to character states outlined in Table S2.

| **Species** | **col.shape** | **diss.tissue** | **connect.elem1** | **pores** | **corallite.shape** | **corallite.diam** |
| --- | --- | --- | --- | --- | --- | --- |
| *Actinotheca tenuicostata* | 2 | 1 | 1 | 1 | 1 | 4 |
| *Adetopora tikhyi* | 2 | 1 | 1 | 1 | 1 | 2 |
| *Alveolitella fecunda* | 4 | 1 | 2 | 3 | 2 | 1 |
| *Alveolitella polygona* | 4 | 1 | 2 | 3 | 2 | 1 |
| *Alveolitella ramosus* | 4 | 1 | 2 | 3 | 4 | 1 |
| *Alveolitella subaequalis* | 4 | 1 | 2 | 3 | 2 | 1 |
| *Alveolites complanatus* | 3 | 1 | 1 | 1 | 3 | 1 |
| *Alveolites compressus* | 3 | 1 | 2 | 3 | 2 | 1 |
| *Alveolites delhayei* | 3 | 1 | 2 | 3 | 3 | 1 |
| *Alveolites densatus* | 4 | 1 | 2 | 3 | 2 | 1 |
| *Alveolites duponti* | 4 | 1 | 2 | 3 | 2 | 1 |
| *Alveolites edwardsi* | 3 | 1 | 2 | 3 | 3 | 1 |
| *Alveolites edwardsi frasnianus* | 3 | 1 | 2 | 3 | 4 | 1 |
| *Alveolites elongatus* | 3 | 1 | 2 | 3 | 3 | 1 |
| *Alveolites gosseleti* | 3 | 1 | 2 | 3 | 3 | 1 |
| *Alveolites indet.* | 3 | 1 | 2 | 3 | 4 | 1 |
| *Alveolites lamellosus* | 3 | 1 | 2 | 3 | 3 | 1 |
| *Alveolites maillieuxi* | 3 | 1 | 2 | 3 | 2 | 1 |
| *Alveolites multispinosus* | 3 | 1 | 2 | 3 | 3 | 1 |
| *Alveolites obtortiformis* | 3 | 1 | 2 | 3 | 2 | 1 |
| *Alveolites obtortus* | 3 | 1 | 2 | 3 | 2 | 1 |
| *Alveolites parvus* | 3 | 1 | 2 | 3 | 3 | 1 |
| *Alveolites regularis* | 3 | 1 | 2 | 3 | 2 | 1 |
| *Alveolites saleei* | 4 | 1 | 2 | 2 | 3 | 1 |
| *Alveolites smithi* | 3 | 1 | 2 | 2 | 4 | 1 |
| *Alveolites* sp. | 3 | 1 | 2 | 3 | 3 | 1 |
| *Alveolites suborbicularis* | 3 | 1 | 2 | 3 | 3 | 1 |
| *Alveolites tenuissimus* | 3 | 1 | 1 | 1 | 3 | 1 |
| *Aulocystis* sp. | 2 | 1 | 1 | 1 | 1 | 4 |
| *Aulopora* cf. *compacta* | 1 | 1 | 1 | 1 | 1 | 2 |
| *Aulopora lata* | 1 | 1 | 1 | 1 | 1 | 3 |
| *Aulopora liniformis* | 1 | 1 | 1 | 1 | 1 | 1 |
| *Aulopora parva* | 1 | 1 | 1 | 1 | 1 | 1 |
| *Aulopora serpens* | 1 | 2 | 1 | 1 | 1 | 2 |
| *Aulopora slosarskii* | 1 | 2 | 1 | 1 | 1 | 2 |
| *Aulopora* sp. *A* | 1 | 1 | 1 | 1 | 1 | 2 |
| *Aulopora* sp. *B* | 1 | 1 | 1 | 1 | 1 | 3 |
| *Aulopora* sp. *C* | 1 | 1 | 1 | 1 | 1 | 2 |
| *Aulostegites hillae* | 1 | 1 | 2 | 1 | 2 | 2 |
| *Caliapora battersbyi* | 3 | 1 | 2 | 3 | 2 | 1 |
| *Caliapora battersbyi minor* | 3 | 1 | 2 | 3 | 2 | 1 |
| *Caliapora* sp. | 3 | 1 | 2 | 3 | 2 | 1 |
| *Caliapora venusta* | 3 | 1 | 2 | 3 | 2 | 1 |
| *Coenites* aff. *variabilis* | 4 | 1 | 2 | 1 | 5 | 1 |
| *Coenites subramosus* | 4 | 1 | 1 | 1 | 5 | 1 |
| *Crassialveolites aff. crassus* | 3 | 1 | 2 | 3 | 3 | 1 |
| *Crassialveolites cavernosus* | 3 | 1 | 2 | 3 | 4 | 1 |
| *Crassialveolites crassus* | 3 | 1 | 2 | 3 | 4 | 1 |
| *Crassialveolites multiperforatus* | 3 | 1 | 2 | 4 | 2 | 1 |
| *Crassialveolites oliveri* | 3 | 1 | 2 | 3 | 4 | 1 |
| *Crassialveolites* sp. | 3 | 1 | 2 | 3 | 2 | 1 |
| *Dendropora explicita* | 2 | 1 | 1 | 1 | 3 | 1 |
| *Favosites eifeliensis* | 3 | 1 | 2 | 2 | 2 | 3 |
| *Favosites robustus* | 3 | 1 | 2 | 2 | 2 | 4 |
| *Favosites saginatus* | 3 | 1 | 2 | 2 | 2 | 3 |
| *Favosites* sp. *1* | 3 | 1 | 2 | 2 | 2 | 4 |
| *Favosites* sp. *2* | 3 | 1 | 2 | 2 | 2 | 3 |
| *Heliolites porosus* | 3 | 1 | 1 | 1 | 1 | 3 |
| *Hillaepora circulipora* | 4 | 1 | 1 | 1 | 2 | 1 |
| *Hillaepora spicata* | 4 | 1 | 2 | 4 | 2 | 2 |
| *Lecomptopora tumefacta* | 4 | 1 | 2 | 2 | 2 | 1 |
| *Maksymilianites polonicus* | 2 | 2 | 3 | 1 | 1 | 7 |
| *Michelinia vinni* | 3 | 1 | 2 | 2 | 2 | 6 |
| *Natalophyllum* cf. *giveticum* | 4 | 1 | 2 | 1 | 2 | 1 |
| *Pachyfavosites polonicus* | 3 | 1 | 2 | 4 | 2 | 1 |
| *Pachyfavosites polymorpha* | 3 | 1 | 2 | 2 | 2 | 2 |
| *Platyaxum clathratum minus* | 5 | 1 | 1 | 1 | 5 | 1 |
| *Platyaxum escharoides* | 5 | 1 | 2 | 3 | 5 | 1 |
| *Plexituba bricae* | 1 | 2 | 1 | 1 | 1 | 3 |
| *Remesia crispa* | 3 | 2 | 3 | 1 | 1 | 3 |
| *Roseoporella gradata* | 3 | 1 | 1 | 1 | 5 | 1 |
| *Roseoporella heuvelmansi* | 3 | 1 | 2 | 3 | 2 | 1 |
| *Roseoporella* sp. *A* | 3 | 1 | 2 | 3 | 2 | 1 |
| *Roseoporella* sp. *B* | 3 | 1 | 2 | 3 | 2 | 1 |
| *Sapounofouskilites minimus* | 2 | 2 | 3 | 1 | 1 | 2 |
| *Scoliopora denticulata* | 4 | 1 | 2 | 2 | 4 | 1 |
| *Scoliopora kaisini* | 4 | 1 | 2 | 3 | 4 | 1 |
| *Scoliopora longispina* | 4 | 1 | 2 | 4 | 3 | 1 |
| *Scoliopora maillieuxi* | 3 | 1 | 2 | 3 | 4 | 1 |
| *Scoliopora* sp. *A* | 4 | 1 | 2 | 3 | 3 | 1 |
| *Scoliopora* sp. *B* | 4 | 1 | 2 | 3 | 3 | 1 |
| *Senceliaepora tenuiramosa* | 4 | 1 | 2 | 1 | 1 | 1 |
| *Squameoalveolites fornicatus* | 3 | 1 | 2 | 3 | 3 | 1 |
| *Striatopora* aff. *peetzi* | 4 | 1 | 2 | 2 | 3 | 1 |
| *Striatopora* aff. *tenuis* | 4 | 1 | 2 | 2 | 2 | 2 |
| *Striatopora enigmatica* | 4 | 1 | 2 | 2 | 2 | 1 |
| *Striatopora sciuricauda* | 4 | 1 | 2 | 2 | 2 | 1 |
| *Syringocystis eifeliensis* | 3 | 2 | 3 | 1 | 1 | 5 |
| *Syringopora* cf. *volkensis* | 2 | 1 | 3 | 1 | 1 | 2 |
| *Syringopora hilarowiczi* | 2 | 2 | 3 | 1 | 1 | 2 |
| *Syringopora kowalensis* | 2 | 2 | 3 | 1 | 1 | 2 |
| *Syringopora* sp. | 2 | 2 | 3 | 2 | 1 | 8 |
| *Syringopora tikhyiformis* | 2 | 1 | 3 | 1 | 1 | 2 |
| *Syringoporella raritabulata* | 2 | 2 | 3 | 1 | 1 | 2 |
| *Syringoporella* sp. | 2 | 2 | 3 | 1 | 1 | 2 |
| *Thamnopora alta* | 4 | 1 | 2 | 2 | 2 | 1 |
| *Thamnopora angusta* | 4 | 1 | 2 | 3 | 2 | 1 |
| *Thamnopora auberti* | 4 | 1 | 2 | 2 | 2 | 2 |
| *Thamnopora beliakovi* | 4 | 1 | 2 | 2 | 2 | 1 |
| *Thamnopora boloniensis* | 4 | 1 | 2 | 2 | 2 | 2 |
| *Thamnopora cervicornis* | 4 | 1 | 2 | 2 | 2 | 3 |
| *Thamnopora* cf. *cylindrica* | 4 | 1 | 2 | 2 | 2 | 1 |
| *Thamnopora* cf. *irregularis* | 4 | 1 | 2 | 2 | 2 | 2 |
| *Thamnopora dubia* | 4 | 1 | 2 | 2 | 2 | 2 |
| *Thamnopora* ex gr. *boloniensis* | 4 | 1 | 2 | 2 | 2 | 2 |
| *Thamnopora fromelennensis* | 4 | 1 | 2 | 2 | 2 | 1 |
| *Thamnopora irregularis* | 4 | 1 | 2 | 1 | 2 | 1 |
| *Thamnopora lecomptei* | 4 | 1 | 2 | 2 | 2 | 2 |
| *Thamnopora legibilis* | 4 | 1 | 2 | 2 | 2 | 1 |
| *Thamnopora micropora* | 4 | 1 | 2 | 2 | 2 | 1 |
| *Thamnopora patula* | 4 | 1 | 2 | 2 | 2 | 2 |
| *Thamnopora polyforata* | 4 | 1 | 2 | 2 | 2 | 1 |
| *Thamnopora proba* | 4 | 1 | 2 | 2 | 2 | 1 |
| *Thamnopora reticulata* | 4 | 1 | 2 | 1 | 2 | 1 |
| *Thamnopora* sp. *A* | 4 | 1 | 2 | 2 | 2 | 1 |
| *Thamnoptychia mistiaeni* | 4 | 1 | 1 | 1 | 2 | 4 |
| *Thecostegites bouchardi* | 3 | 2 | 3 | 1 | 1 | 2 |
| *Thecostegites dumoni* | 3 | 1 | 3 | 1 | 1 | 2 |
| *Thecostegites lepas* | 3 | 1 | 3 | 1 | 1 | 1 |
| *Thecostegites major* | 2 | 2 | 3 | 1 | 1 | 2 |
| *Vaughania* cf. *geometrica* | 3 | 1 | 2 | 4 | 2 | 2 |
| *Yavorskia paszkowskii* | 3 | 1 | 2 | 4 | 2 | 7 |
| *Yavorskia* sp. *1* | 3 | 2 | 2 | 4 | 2 | 4 |

**Table S2.** Morphological characters, character states, and interpretations of ecological significance of each morphological character. Additional references not cited in the main text are provided below.

| **Morphological Character** | **Character States** | **Interpretation** | **Source** |
| --- | --- | --- | --- |
| dissepimental tissue | 1-absent, 2-present | Feeding strategy, sediment rejection. Dissipmental tissue present indicates adaptation to turbid environments | Berkowski 2012, Sorauf 2007 |
| connecting elements | 1-absent, 2-pores; 3-tubuli | Increasing colony integration and likelihood of photosymbiosis | Hughes 1987  [11, 33,36,37]. |
| pore location | 1-absent, 2-wall, 3-corners, 4 both | Colony-wide coordinated response to stress, likelihood of photosymbiosis, | Swain et al. 2018  [33,36,37]. |
| corallite shape | 1-round; 2-subpolygonal/polygonal; 3-elongated; 4-meandroid, 5-crescentic | Colony integration |  |
| corallite diameter | 1 – small (<1mm), 2 – medium (1-2mm), 3 –large (>2mm) | Likelihood of photosymbiosis as only photosymbiotic corals possess small corallites. | Porter et al. 1976 |

**References**

Berkowski, B. 2012. Life strategies and function of dissepiments in rugose coral *Catactotoechus instabilis* from the Lower Devonian of Morocco. Acta Palaeontologica Polonica 57 (2): 391–400.

Hughes, T. P. (1987). Skeletal density and growth form of corals. *Marine Ecology Progress Series*, 35, 259-266.

Porter, J.W. (1976) Autotrophy, heterotrophy, and resource partitioning in Caribbean reef-building corals. *The American Naturalist* 110:731–742.

Sorauf, J. E. 2003. The function of dissepiments and marginaria in the Rugosa (Cnidaria, Zoantharia). *Fossil Corals and Sponges, Proceedings of the 9th International Symposium on Fossil Cnidaria and Porifera, Graz*, 11-29.

Swain, T. D., Bold, E. C., Osborn, P. C., Baird, A. H., Westneat, M. W., Backman, V., & Marcelino, L. A. (2018). Physiological integration of coral colonies is correlated with bleaching resistance. *Marine Ecology Progress Series*, 586, 1-10.
